# Design of the MERCURION-IPF trial – intravenous immunoglobulin for the treatment of acute exacerbations of idiopathic pulmonary fibrosis

**DOI:** 10.3389/fimmu.2026.1777564

**Published:** 2026-05-05

**Authors:** Vasilina Sotiropoulou, Fotios Sampsonas, Eirini Vasarmidi, Katerina Antoniou, Athena Gogali, Konstantinos Kostikas, Fotios Drakopanagiotakis, Paschalis Steiropoulos, George Margaritopoulos, Konstantinos Porpodis, Zoe Daniil, Ioannis Tomos, Ilias Papanikolaou, Vasilios Tzilas, Stelios Loukides, Argyrios Tzouvelekis

**Affiliations:** 1 Department of Respiratory Medicine, University Hospital of Patras, Patras, Greece; 2 Laboratory of Molecular and Cellular Pulmonology, Department of Respiratory Medicine, University of Crete, Heraklion, Greece; 3 Department of Respiratory Medicine, University Hospital of Ioannina, Ioannina, Greece; 4 Department of Respiratory Medicine, Medical School, Democritus University of Thrace, Alexandroupolis, Greece; 5 Pulmonary Department, George Papanikolaou Hospital, Aristotle University of Thessaloniki, Thessaloniki, Greece; 6 Department of Respiratory Medicine, University Hospital of Larissa, Larissa, Greece; 7 5th Pulmonary Department, ‘SOTIRIA’ Chest Diseases Hospital of Athens, Athens, Greece; 8 Pulmonary Department, Corfu General Hospital, Corfu, Greece; 9 2nd Respiratory Medicine Department, Attikon University Hospital, National and Kapodistrian University of Athens, Athens, Greece; 10 Department of Internal Medicine, Pulmonary, Critical Care and Sleep Medicine, Yale School of Medicine, New Haven, CT, United States

**Keywords:** acute exacerbation of idiopathic pulmonary fibrosis, clinical trial, idiopathic pulmonary fibrosis, intravenous immunoglobulin, protocol

## Abstract

**Introduction:**

Despite their profound clinical impact and high mortality, acute exacerbations of idiopathic pulmonary fibrosis (AE-IPF) still lack any effective or standardized treatment strategies. This manuscript describes the rationale and design of a randomized, multicenter, open-label Phase III clinical trial evaluating the efficacy of intravenous immunoglobulin (IVIG) compared with usual care in hospitalized patients with AE-IPF.

**Methods:**

This trial will enroll 196 patients across eight different sites in Greece. Inclusion criteria are designed to identify patients with AE-IPF according to the American Thoracic Society definition, while excluding those with cardiac decompensation or pulmonary embolism. The primary endpoint is a composite of all-cause in-hospital mortality or the need for intubation. Secondary endpoints include all-cause mortality at 30 and 90 days, hospital readmission or a new AE-IPF episode within 90 days, and the change in the PaO_2_/FiO_2_ ratio from hospital admission to discharge.

**Discussion:**

Based on the concept that patients with IPF frequently demonstrate impaired cellular and humoral immunity, and inflammation and/or immune dysregulation may contribute to AE-IPF pathogenesis, there is a strong rationale for evaluating the therapeutic usefulness of IVIG in this setting. Retrospective data indicate that IVIG could provide clinical benefit in AE-IPF, potentially through its anti-inflammatory and immunomodulatory effects. In this trial, IVIG will be administered as an adjunct to usual care, which includes pulse corticosteroids, broad-spectrum antibiotics, prophylactic anticoagulation, and oxygen therapy. This intervention has the potential to significantly influence current treatment strategies for AE-IPF.

**Clinical trial registration:**

ClinicalTrials.gov, identifier NCT05745545.

## 1 Introduction

Acute exacerbation of idiopathic pulmonary fibrosis (AE-IPF) is defined as an acute worsening of patient’s respiratory status, characterized by marked respiratory symptoms and appearance of new bilateral alveolar abnormalities, which cannot be fully explained by cardiac decompensation or fluid overload (1, 2). AE-IPF carries a grave prognosis, with in-hospital mortality exceeding 50% and rising to 75–90% among patients requiring mechanical ventilation. For those who survive hospitalization, the median survival after an AE-IPF is only 3–4 months (3–5). AE-IPF is classified as either idiopathic, when no precipitating factor can be identified, or triggered, when associated with identifiable causes, such as infection, gastroesophageal reflux, aspiration, drug toxicity, lung biopsy, or other invasive procedures or surgical interventions (1, 6, 7).

The pathogenic mechanisms of AE-IPF have not been fully elucidated, preventing rational development of targeted therapies. Excessive epithelial cell apoptosis, aberrant inflammation and immune dysregulation are thought to underlie the events of this devastating condition. Elevated levels of pro-inflammatory cytokines, high cellularity of the bronchoalveolar lavage fluid and histopathological findings of diffuse alveolar damage are highly suggestive of an aberrant inflammatory response, interplayed with exuberant alveolar cell apoptosis (8–10). Inflammatory changes may extend beyond the lungs with systemic consequences and multi-organ dysfunction (8, 11, 12). Moreover, recent evidence highlights the role of antibodies in the pathogenesis of AE-ILDs through various mechanisms, including autoimmunity, complement activation and direct cytotoxicity leading to alveolitis and endothelitis (13–15).

Well-established treatment strategies for AE-IPF are largely lacking, and substantial heterogeneity persists in clinical practice. The latest IPF guidelines propose the use of corticosteroids in AE-IPF, despite limited and controversial supporting evidence (16). Importantly, in a recently published global survey, the majority of physicians across the world apply pulses of methylprednisolone (250 mg daily for 3–5 days) in those cases (3). Recently, the EXAFIP trial showed that the administration of intravenous cyclophosphamide in addition to corticosteroid pulses was associated with increased 3-month mortality in patients with AE-IPF; yet, the enhanced toxic profile was largely attributed to the synergistic immunosuppressive effects of the combined corticosteroid and cyclophosphamide administration (17). To this end, two clinical trials are currently ongoing in AE-IPF: EXAFIP2 (NCT05674994), evaluating corticosteroids versus placebo, with all-cause 30-day mortality as the primary outcome, and STRIVE-IPF (NCT03286556) investigating triple autoantibody reduction therapy—therapeutic plasma exchange, rituximab, and intravenous immunoglobulin (IVIG)—with six-month survival as the primary endpoint.

## 2 Methods and analysis

### 2.1 Trial design

This is a randomized, multicenter, open-label Phase III clinical trial to determine the efficacy of IVIG, in comparison to usual treatment, among patients with AE-IPF. A total of 196 subjects (power analysis see below) will be enrolled among eight sites in Greece. The open-label design was selected due to feasibility considerations and the acute clinical setting; to minimize potential bias, predefined objective outcome measures will be used, and data collection and outcome assessment will follow standardized criteria.

**Figure 1:**
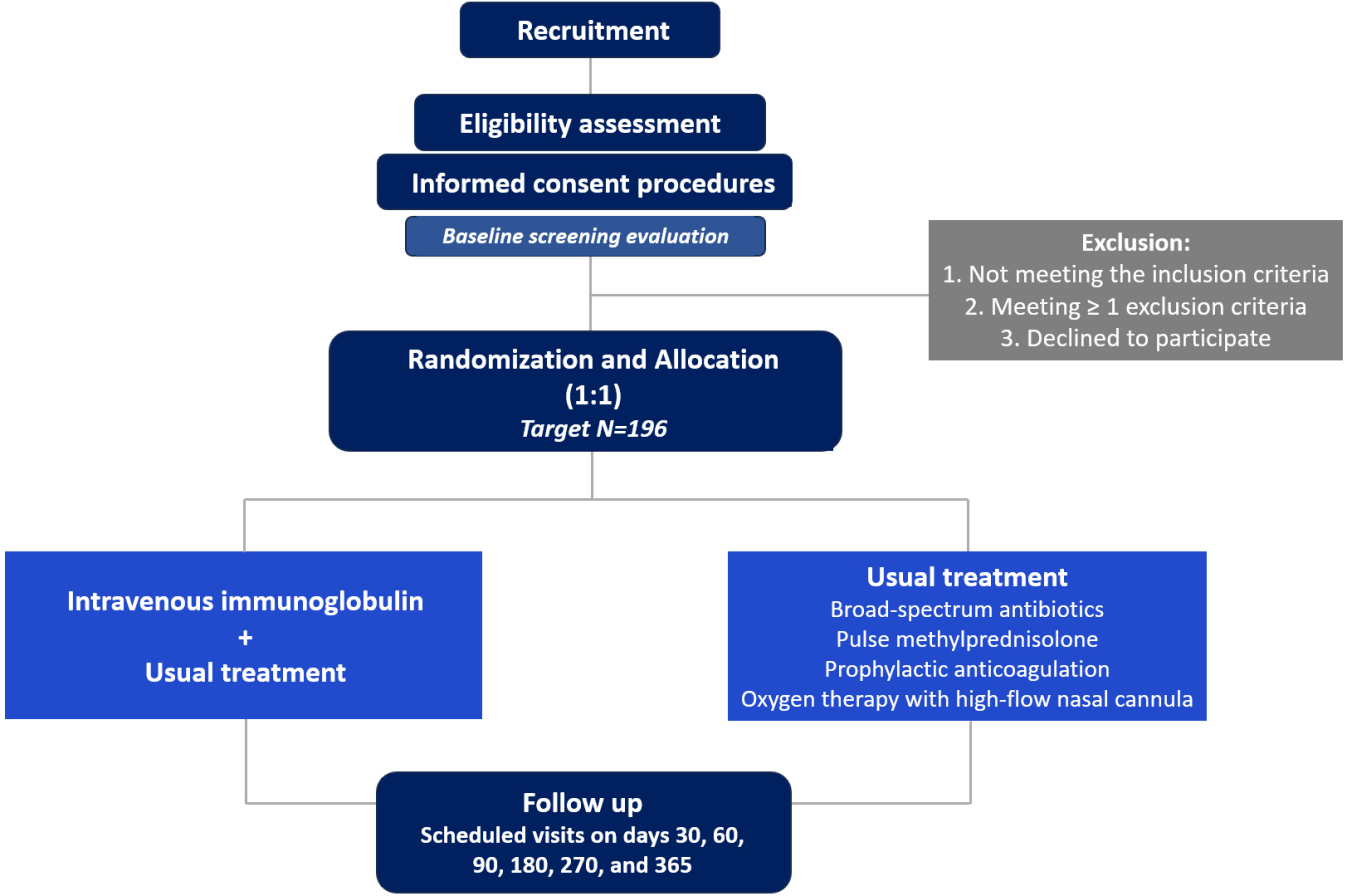
Study protocol flow diagram.

Subjects will be recruited from the inpatient populations of the collaborating institutions. Potential participants will initially be identified by the attending physician or clinical care team, who may include or work alongside the study investigators. The clinical team will then seek the patient’s consent to permit the study investigators to approach them and provide detailed information about the research project. Upon completion of informed consent and baseline/screening assessments, eligible participants will be randomized in a 1:1 ratio to one of two treatment arms: IVIG (experimental therapy) in addition to standard care versus usual care alone. Usual treatment consists of empiric broad-spectrum antibiotics, pulse methylprednisolone, prophylactic anticoagulation, and oxygen therapy with high-flow nasal cannula, as outlined below, and will be provided to all participants, including those allocated to the experimental arm. A schematic representation of the study protocol and planned participant flow is provided in **Figure 1**.

### 2.2 Study population

The trial’s inclusion criteria focus on identifying individuals who meet the American Thoracic Society definition of AE-IPF (1), while excluding those with cardiac decompensation, pulmonary embolism, or conditions that increase procedural risk. A detailed overview of the eligibility criteria is provided in **Table 1**.

**Table 1:** Study eligibility criteria.

Inclusion criteria
Patients ≥ 18 years of age Patients with IPF diagnosis that fulfils ATS/ERS Consensus Criteria (16). Patients hospitalised with a definite or suspected AE-IPF diagnosis, as defined by the international working group criteria (1) and as ascertained by the responsible Primary Investigator.
The criteria of IPF-AE are as follows: - Previous or concurrent diagnosis of IPF - Acute worsening or development of dyspnoea typically < 1-month duration - Computed tomography with new bilateral ground-glass opacity and/or consolidation superimposed on a background pattern consistent with usual interstitial pneumonia pattern - Deterioration not fully explained by cardiac failure or fluid overload
Patients who fail to meet all 4 criteria due to missing computed tomography should be considered as having "suspected Acute Exacerbation". If the diagnosis of IPF is not previously established, this criterion can be met by the presence of radiologic and/or histopathologic changes consistent with usual interstitial pneumonia pattern on the current evaluation. If no previous computed tomography is available, the qualifier "new" can be dropped from the third AE-IPF criterion.
4. Patient able to understand and sign a written informed consent form. In case of incapacity of the patient, the written informed consent form will be signed by the patients’ legally authorized representative.
Exclusion criteria
Patients with acute worsening due to uncontrolled heart failure or pulmonary embolism. Patients with known hypersensitivity to corticosteroids, IVIG or any component of the study treatment. Patients with known IgA deficiency (IgA level <7 mg/dL)- to preclude IVIG reactions. Patients without a definite diagnosis of IPF or AE-IPF based on clinical, radiological, laboratory evaluation, and multidisciplinary discussion. Patients with active malignancy or currently receiving cancer treatment, except for basal cell or squamous cell skin cancer or low-risk prostate cancer (T1 or T2a stage with PSA <10 ng/dL). These criteria are aligned with current guidelines. Patients that have received treatment for >14 days within the preceding month with >20mg daily prednisone (or equivalent) or any treatment during the last month with immunosuppressants (e.g., cyclophosphamide, mycophenolate etc.) according to already published therapeutic protocols or > 1 mg/kg/d from more than 7 days in the last 15 days. Patients participating to another interventional clinical trial. Patients with documented pregnancy or lactation. Patients under tutorship or curatorship. Patients deprived of liberty or under court protection. Patients who refuse to participate or decline to provide written informed consent.

### 2.3 Patient evaluation

Patients who meet the inclusion criteria and do not have any exclusion criteria will undergo a comprehensive evaluation upon presentation to the emergency department. The initial assessment will include a computed tomography scan with pulmonary artery contrast protocol and echocardiography. In patients with severe renal impairment (glomerular filtration rate <15 ml/min), contrast administration will be avoided, and these patients will be excluded from the study, as pulmonary embolism cannot be reliably ruled out using a ventilation-perfusion scan. Laboratory evaluation will include complete blood count, biochemical profile, coagulation tests, C-reactive protein, erythrocyte sedimentation rate, procalcitonin, quantitative serum immunoglobulin levels, urinalysis, and cultures of blood, urine, and sputum, as well as rapid testing for SARS-CoV-2 and influenza. When feasible and clinically appropriate, bronchoscopy with collection of bronchoalveolar lavage fluid will be performed for microbiological analysis. Potential triggers of AE-IPF, both known and idiopathic, will be systematically investigated and recorded.

### 2.4 Data collection

Baseline demographic data (including age, sex, and smoking status) and clinical characteristics (including comorbidities, administered oxygen therapy, and treatment regimens) will be systematically collected. Functional and imaging parameters will be recorded before, during, and after hospitalization, along with treatment-related adverse events and overall hospitalization outcomes. Biological samples, including serum and plasma obtained after centrifugation or bronchoalveolar lavage fluid in cases where bronchoscopy is performed, will be collected for biomarker studies. A separate informed consent form will be provided for this procedure, and participation in the study will not be affected if a participant chooses not to provide biological samples.

### 2.5 Study endpoints

The primary endpoint of this trial is all-cause in-hospital mortality or intubation. Key clinical secondary endpoints include 30-day and 90-day all-cause mortality, hospital readmission or new AE-IPF within 180 days and change in PaO_2_/FiO_2_ ratio from hospital admission to discharge. Patients discharged from the hospital will be instructed to return for follow-up visits as per the scheduled assessments. Adverse events and complications that occur anytime during patients’ hospitalization will be recorded and compared. Exploratory outcomes include assessing changes in forced vital capacity and diffusing capacity for carbon monoxide at 90 days after discharge.

### 2.6 Randomization and sample size

The study will use 1:1 randomization (experimental arm versus usual treatment arm), with patients assigned to a group based on the time of presentation to the emergency department. Simple sequential randomization without blocks will be employed, and no site stratification will be performed. The randomization process will be conducted centrally in advance by the Data Coordinating Center (DCC) using a secure, computer-based system to ensure allocation concealment and maintain study integrity. Stratified randomization was not implemented due to anticipated recruitment challenges, in order to avoid small numbers within individual strata and unnecessary complexity in the randomization process.

The sample size was calculated assuming a 1:1 randomization between the IVIG plus usual treatment and usual treatment groups, a two-sided chi-square test, a type I error rate (α) of 0.05, and 80% power. Based on existing literature, the expected rate of the primary endpoint (death or intubation) was 70% in the usual care group (3–5) and 50% in the IVIG plus usual care group (18, 19). This yielded an initial sample size of 93 patients per group. Allowing for a 5% dropout rate, the final target enrollment was set at 98 patients per group, for a total of 196 patients.

### 2.7 Study drug administration

#### 2.7.1 Experimental therapy - intravenous immunoglobulin

IVIG will be administered at a total dose of 1 g/kg, divided over three consecutive days. The IVIG dosage is derived from our previous study and represents the high-dose regimen required to achieve its immunomodulatory and anti-inflammatory effects (18, 20). This regimen is consistent with ACR and CHEST guidelines for ILDs associated with systemic autoimmune diseases (21), while published data in this setting remain limited. Infusion will start at a rate of 0.5 mg/kg/hour for the first 15 minutes and, if no adverse reaction occurs, the rate will then be increased step-wise as tolerated. Participants will be premedicated with acetaminophen and levocetirizine.

Adverse events related to IVIG administration will be systematically assessed during each infusion and throughout the study period. Major events—such as thromboembolic complications, hemolysis, renal dysfunction, arrhythmias, significant fever, hemodynamic instability, and fluid overload— or infusion reactions will be monitored through vital signs, fluid balance, and relevant laboratory tests. Computed tomography Pulmonary Angiogram (CTPA) and lower limb compression ultrasound (CUS) as well as cardiac ultrasound will be performed upon treating physician’s clinical suspicion to exclude cases of thromboembolism following IVIG infusion. All events will be documented, graded by severity, and managed according to institutional safety protocols.

#### 2.7.2 Usual treatment

Usual treatment will consist of empiric broad-spectrum antibiotics, pulse methylprednisolone, prophylactic anticoagulation, and oxygen therapy with high-flow nasal cannula and will be provided to all participants, at all sites, in both treatment arms.

##### 2.7.2.1 Corticosteroids

All participants will receive a pulse regimen of intravenous methylprednisolone at 250 mg daily from day 1 to day 3, with no additional corticosteroids administered thereafter.

Current evidence is insufficient to establish the optimal dose and duration of corticosteroid therapy in patients with AE-IPF. Retrospective data from AE-IPF cohorts suggest no significant difference in in-hospital mortality between high-dose (500–1000 mg/day for 3 days) and low-dose (100–200 mg/day for 5 days) pulse methylprednisolone regimens while early corticosteroid tapering was associated with a more favorable prognosis (22–24). Accordingly, and considering that pulse regimens maximize immunomodulatory and anti-inflammatory effects while minimizing the adverse effects and immunosuppression associated with prolonged corticosteroid use (25), our trial will employ this corticosteroid dosing schedule.

##### 2.7.2.2 Antibiotics

All participants will receive empirical broad-spectrum antibiotics within the first 24 hours of hospitalization. Antibiotic regimens, will be standardized whenever possible and systematically recorded, with type, dose, and duration documented for inclusion in the statistical analysis to adjust for potential confounding effects. The initial regimen will include a respiratory quinolone and/or an antipseudomonal penicillin, if indicated. Given the challenges in confirming infections in these often critically ill patients, empiric antibiotic therapy is widely accepted and recommended (1, 2). This empirical approach aims to cover common pathogens implicated in AE-IPF and potential colonization with *Pseudomonas* spp. or *Haemophilus influenzae*, taking into account the frequent hospitalizations and structural lung changes seen in IPF patients, as well as existing evidence on other patients with severe lower respiratory tract infections (26–28). The duration or escalation of antibiotic therapy may be adjusted based on available antibiograms or the treating physician’s clinical judgment; any modifications should be documented and justified.

##### 2.7.2.3 Anticoagulation

All patients will receive prophylactic-dose anticoagulation (low molecular weight heparin or fondaparinux) throughout hospitalization, in accordance with current guidelines for thromboprophylaxis in critically ill patients (29). Patients with an established indication for therapeutic anticoagulation will be maintained on their therapeutic regimen.

##### 2.7.2.4 Antifibrotic therapy

All patients who are receiving antifibrotic therapy (nintedanib, pirfenidone, or nerandomilast) at the time of AE-IPF diagnosis will continue treatment during hospitalization, unless contraindicated. This is supported by evidence indicating that both nintedanib and pirfenidone may be beneficial in AE-IPF, with nintedanib shown to delay time to first exacerbation and pirfenidone to reduce respiratory-related hospitalizations (30–33). Patients not previously on antifibrotic therapy will not initiate treatment during hospitalization. This approach aligns with current clinical practice, as most physicians prefer to start antifibrotic therapy after clinical stabilization (3).

### 2.8 Study duration and follow-up

Participants may be discharged from the hospital when deemed clinically appropriate by their attending physicians. Individuals withdrawn due to intolerable adverse events will continue to be monitored until the event resolves or reaches a stable condition. Participants discharged from inpatient care will be asked to return for scheduled follow-up visits on days 30, 60, 90, 180, 270, and 365. Additional telephone contacts will be conducted by study staff at the remaining monthly intervals to identify delayed complications and assess ongoing outcomes.

### 2.9 Statistical analysis

Patients’ baseline demographic and clinical characteristics will be presented as means with standard deviations or medians with interquartile ranges for continuous variables, and as counts and percentages for categorical measures. Normality of distribution will be examined using Kolmogorov–Smirnov test. Group comparisons, to assess the effectiveness of randomization, will be performed using t-tests for continuous variables and Fisher’s exact test for categorical variables. Survival analyses be summarized using Kaplan Meier plots. All analyses will be conducted on an intention-to-treat basis. Two-sided p-values <0.05 will be considered statistically significant.

Predefined subgroup analyses will be conducted for patients with low baseline IgG levels, if the sample size allows, to evaluate whether IgG deficiency modifies the effect of IVIG on clinical outcomes. In addition, subgroup analyses will be performed for major trigger categories, and relevant baseline characteristics will be included in statistical models to account for heterogeneity.

## 3 Discussion

### 3.1 Rationale for trial design

This clinical trial is motivated by the potential benefits of IVIG in AE-IPF, due to its anti-inflammatory and immunomodulatory effects (34) and its proposed use in several forms of ILDs (21). Specifically, IVIG has an established role in rapidly progressive ILDs and as second-line therapy for ILDs associated with idiopathic inflammatory myopathies and mixed connective tissue disease, particularly when infection is a major concern (21). IVIG exerts pleiotropic immunomodulatory effects. These include blockade of the neonatal Fc receptor, leading to reduced IgG recycling and circulating IgG levels, as well as neutralization of pathogenic antibodies, inhibition of antibody-mediated cytotoxicity and natural killer cell activity, and suppression of autoantibody production and complement activation (35). Considering that patients with IPF often exhibit impaired cellular and humoral immunity (12, 15, 36, 37), and that dysregulated inflammatory and immune responses play major role in AE-IPF pathogenesis (8), there is a strong rationale to investigate the use of IVIG in this setting. Our prior retrospective observational study provided preliminary evidence that IVIG could serve as a potentially effective adjunct to usual therapies, as it seemed to be associated with improved gas exchange and improved survival in a significant proportion of treated patients (n=39) hospitalized with acute exacerbation of fibrotic interstitial lung disease, including IPF (18). Those findings were consistent with other previously published studies (19).

In this study, IVIG will be administered as an adjunct to usual care, which will include pulse corticosteroids, broad-spectrum antibiotics, prophylactic anticoagulation, and oxygen therapy. Outcomes will be compared with patients receiving usual treatment alone. Despite the debated efficacy of corticosteroids in AE-IPF and the limited, low-quality evidence available so far, their use is endorsed by treatment guidelines and remains widespread in clinical practice (3, 16). In light of the complex, multi-pathway pathogenesis of AE-IPF and the need for a multi-targeted, oncologic therapeutic approach (8, 11–15) and the proven synergistic effects of IVIG combined with corticosteroids in other diseases (e.g., Kawasaki disease, idiopathic thrombocytopenic purpura, and toxic epidermal necrolysis/Stevens–Johnson syndrome) (38–40) and in order to avoid depriving patients of corticosteroids—a potentially effective treatment currently under investigation (EXAFIP2, NCT05674994) —we decided to administer corticosteroids as usual treatment to both study arms. Corticosteroids will be administered in low-dose pulse regimens and abruptly discontinued after 3 days without tapering, because studies have shown that tapering and long-term administration are the two factors that cause greater immunosuppression in patients and lead to harmful effects (22–25).

### 3.2 Rationale for study endpoints

The primary endpoint of this trial is a composite of all-cause in-hospital mortality or the need for intubation. These events are unequivocal and objective, allowing for accurate and consistent assessment across all participating centers. The use of this composite endpoint reflects the severe and rapidly progressive nature of AE-IPF, capturing the most clinically relevant outcomes and providing a robust measure of treatment efficacy. Secondary endpoints offer further insight into both short- and medium-term patient outcomes, including all-cause mortality at 30 and 90 days, hospital readmission, or the occurrence of a new acute exacerbation of idiopathic pulmonary fibrosis. They also capture indicators of clinical improvement related to treatment, as reflected by changes in the PaO_2_/FiO_2_ ratio from hospital admission to discharge.

In conclusion, acute exacerbation represents a devastating event in the course of IPF, for which no therapy has yet shown a definitive clinical benefit. This prospective, multicenter, randomized study seeks to address this critical unmet need by evaluating the efficacy of IVIG in patients with AE-IPF. We anticipate that the results will provide valuable clinical insights and help guide the management of this life-threatening condition.
